# An *In Vivo* Selection Identifies *Listeria monocytogenes* Genes Required to Sense the Intracellular Environment and Activate Virulence Factor Expression

**DOI:** 10.1371/journal.ppat.1005741

**Published:** 2016-07-14

**Authors:** Michelle L. Reniere, Aaron T. Whiteley, Daniel A. Portnoy

**Affiliations:** 1 Department of Molecular and Cell Biology, University of California, Berkeley, Berkeley, California, United States of America; 2 Graduate Group in Infectious Diseases and Immunity, School of Public Health, University of California, Berkeley, Berkeley, California, United States of America; 3 School of Public Health, University of California, Berkeley, Berkeley, California, United States of America; University of Pennsylvania, UNITED STATES

## Abstract

*Listeria monocytogenes* is an environmental saprophyte and facultative intracellular bacterial pathogen with a well-defined life-cycle that involves escape from a phagosome, rapid cytosolic growth, and ActA-dependent cell-to-cell spread, all of which are dependent on the master transcriptional regulator PrfA. The environmental cues that lead to temporal and spatial control of *L*. *monocytogenes* virulence gene expression are poorly understood. In this study, we took advantage of the robust up-regulation of ActA that occurs intracellularly and expressed Cre recombinase from the *actA* promoter and 5’ untranslated region in a strain in which *loxP* sites flanked essential genes, so that activation of *actA* led to bacterial death. Upon screening for transposon mutants that survived intracellularly, six genes were identified as necessary for ActA expression. Strikingly, most of the genes, including *gshF*, *spxA1*, *yjbH*, and *ohrA*, are predicted to play important roles in bacterial redox regulation. The mutants identified in the genetic selection fell into three broad categories: (1) those that failed to reach the cytosolic compartment; (2) mutants that entered the cytosol, but failed to activate the master virulence regulator PrfA; and (3) mutants that entered the cytosol and activated transcription of *actA*, but failed to synthesize it. The identification of mutants defective in vacuolar escape suggests that up-regulation of ActA occurs in the host cytosol and not the vacuole. Moreover, these results provide evidence for two non-redundant cytosolic cues; the first results in allosteric activation of PrfA via increased glutathione levels and transcriptional activation of *actA* while the second results in translational activation of actA and requires *yjbH*. Although the precise host cues have not yet been identified, we suggest that intracellular redox stress occurs as a consequence of both host and pathogen remodeling their metabolism upon infection.

## Author Summary

Upon recognition of the host, bacterial pathogens activate a genetic virulence program to establish their replicative niche. In this study, we selected for mutants in the model intracellular pathogen *Listeria monocytogenes* that did not up-regulate virulence factors during infection. The screen identified genes involved in sensing the host cell and suggests a model in which expression of virulence factors is spatially and temporally compartmentalized via regulation of transcription and translation. Specifically, results described here indicate two non-redundant host cytosolic cues are sensed by the bacterium in order to activate its virulence program. Future research will illuminate the exact molecular identity of these cytosolic signals. However, the majority of the genes identified are part of the bacterial redox stress response, suggesting that redox changes represent one of the biological cues sensed by *L*. *monocytogenes* to regulate its virulence program.

## Introduction

Intracellular pathogens such as *Plasmodium* spp., *Mycobacterium tuberculosis*, *Salmonella enterica*, *Trypanosoma cruzi*, *and Leishmania* spp. are responsible for an overwhelming amount of morbidity and mortality worldwide. Successful dissemination of many of these pathogens requires complex life cycles that involve survival and replication in environmental or vector niches. To propagate within their hosts, these pathogens establish a variety of unique intracellular niches that are essential for their pathogenesis [1]. Although there is considerable understanding of how intracellular pathogens manipulate host cell biology to promote their pathogenesis, less is known about the precise mechanisms by which these pathogens sense their host cell. Such an understanding may lead to targets for therapeutic intervention. In this study we used *Listeria monocytogenes* as a model system for understanding virulence gene regulation of a facultative intracellular bacterium that transitions from extracellular to intracellular growth.

*L*. *monocytogenes* is a ubiquitous environmental saprophyte capable of causing severe disease as a foodborne pathogen [2]. *L*. *monocytogenes* is also a model system for studying bacterial adaptation to the host [3]. The bacterial virulence program is coordinated with a life cycle that begins upon entry into a mammalian cell either by phagocytosis or bacteria-mediated internalization. To commence intracellular growth, *L*. *monocytogenes* must first escape from the hostile phagosomal environment by the expression and secretion of a cholesterol-dependent cytolysin, listeriolysin O (LLO) that mediates destruction of the phagosome [4]. Upon entry into the cytosol, *L*. *monocytogenes* grows rapidly and expresses an essential determinant of pathogenesis, ActA, an abundant surface protein that mediates host actin polymerization [5,6]. Appropriate regulation of LLO and ActA is critical for *L*. *monocytogenes* pathogenesis and transcriptionally coordinated by the master virulence regulator PrfA [7].

PrfA is a cAMP receptor protein (Crp) family transcriptional regulator that is absolutely essential for *L*. *monocytogenes* virulence gene expression and pathogenesis [8]. PrfA-mediated gene expression is regulated by PrfA abundance, affinity for target promoters, and activation via cofactor binding [9]. PrfA levels are controlled by three promoters. The most proximal promoter contains a site of negative regulation, while the most distal is a PrfA-dependent read-through transcript that is essential for appropriately high levels of intracellular gene expression [10–12]. PrfA binds a palindromic DNA sequence (PrfA-box) and deviations from a consensus sequence result in lower affinity DNA-PrfA interactions [13]. The affinity of PrfA for DNA determines the degree of transcriptional activation prior to PrfA allosteric activation [14]. For example, the gene encoding LLO (*hly*) has a high affinity PrfA-box and consequently is expressed even during growth in broth when PrfA is not activated. In contrast, the *actA* promoter contains a lower affinity PrfA box and is not expressed during growth in broth [15,16]. Upon entry into the host cell cytosol, PrfA is over-expressed and is activated by a two-step process: first, binding of PrfA to DNA requires reduction of the four PrfA cysteine residues while full transcriptional activation of PrfA requires allosteric binding to glutathione [17]. The requirement for glutathione can be bypassed by mutations that lock PrfA in its active conformation (PrfA*) [18]. Strains with PrfA* mutations constitutively express PrfA-activated genes and consequently have growth defects extracellularly, demonstrating the importance of regulating virulence gene expression [19,20]. However, even PrfA* strains grown in broth fail to synthesize the amount of ActA observed intracellularly, which is likely attributable to translational control localized to the 5’ untranslated region (5’ UTR) [21]. Despite these findings of exquisite gene regulation, little is known about trans-acting factors that affect expression of PrfA or PrfA-activated genes.

In a previous study, a genetic system was designed to select for *L*. *monocytogenes* mutants that failed to express ActA intracellularly [17]. This screen led to the identification of *L*. *monocytogenes* glutathione synthase (GshF) and glutathione, a tripeptide antioxidant, as the allosteric activator of PrfA. In this study we sought to further understand the host cues that are recognized by intracellular pathogens during infection. We returned to the forward genetic selection and exhaustively screened for additional mutants that failed to express sufficient ActA intracellularly. This selection identified genes required at each stage of the intracellular lifecycle, including: vacuolar escape, PrfA activation, and cell-to-cell spread. These data suggest a model of compartmentalized gene expression, furthering our understanding of the *L*. *monocytogenes* virulence program.

## Results

### Genetic selection in macrophages

**Fig 1:**
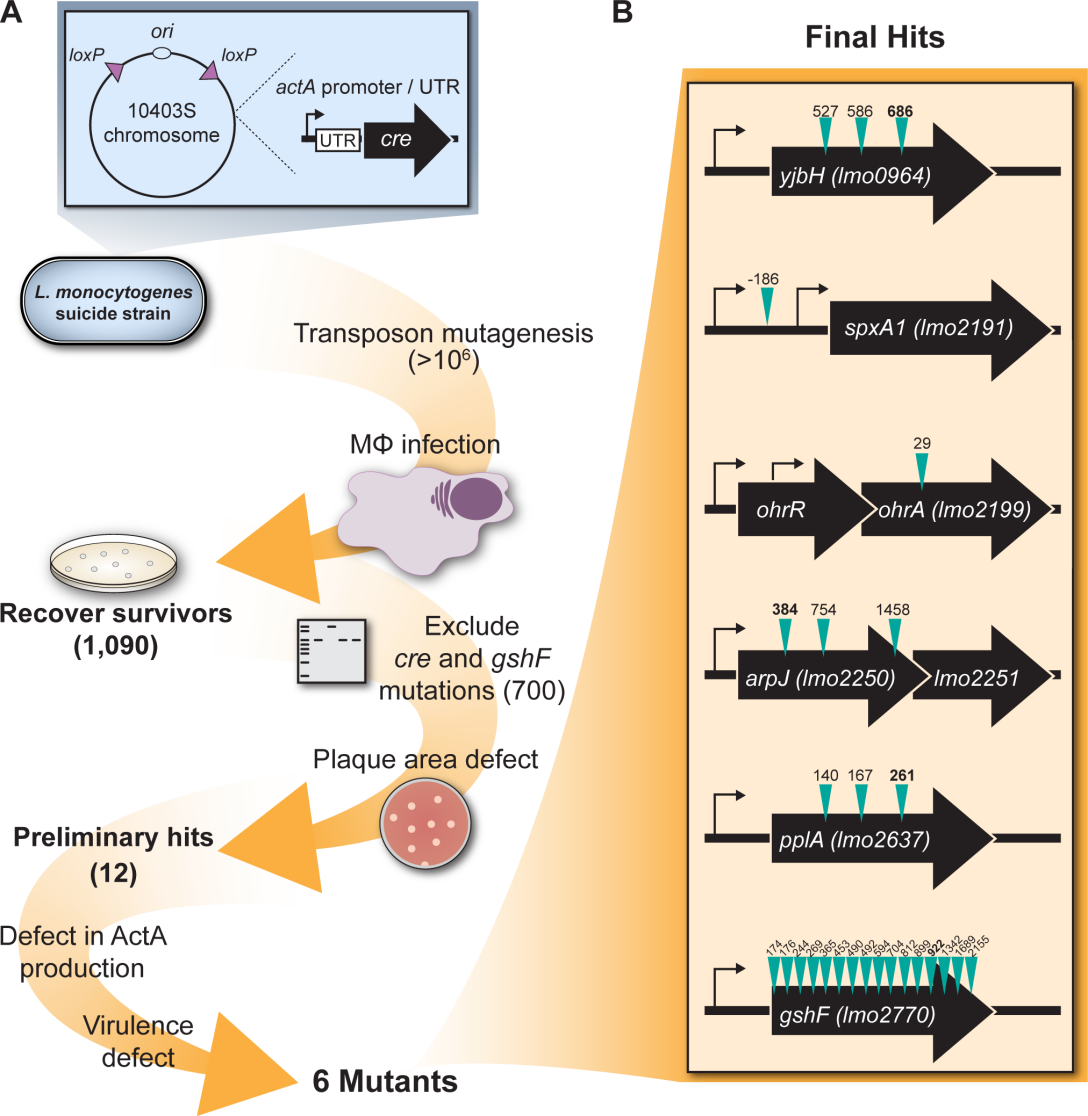
Schematic of genetic selection. **(A)** Description of the genetic selection. Numbers in parentheses indicate number of mutants remaining after each step. See text for more details. **(B)** Genomic context of the genes identified in the selection. Thin black arrows represent predicted transcription start sites [66], teal arrows represent sites of transposon insertions, and numbers above these arrows correspond to mapped transposon locations (nucleotides 3’ of the start codon). Bolded numbers denote the transposon insertions used in this study.

The goal of this study was to identify genes involved in regulation of a principle virulence determinant in *L*. *monocytogenes*, ActA. A bacterial strain was constructed that failed to replicate upon activation of the *actA* gene, which is specifically up-regulated during cytosolic growth and is essential for pathogenesis. This ‘suicide’ strain harbored *loxP* sites in the chromosome flanking the origin of replication (ori) and several essential genes. Codon-optimized *cre* recombinase was expressed from the *actA* promoter (Fig 1A). The suicide strain grew like wild type in rich media but was unrecoverable after infection of bone marrow-derived macrophages (BMMs). A *himar1* transposon library was then constructed in the suicide strain background and used to infect BMMs. When bacteria were isolated at five hours post-infection (p.i.) nearly all mutants harbored transposon insertions in *cre*, the *actA* promoter driving *cre* expression (*actA1p*), *loxP* sites, and *gshF*, encoding glutathione synthase. To identify additional genes required during infection, colonies were isolated at three and four hours p.i, generating a library of 1,090 transposon mutants from an initial inoculum of >1 million bacteria. Colony PCR excluded strains with transposon insertions in *cre* and *gshF*, resulting in a collection of ~700 strains (Fig 1A).

**Fig 2:**
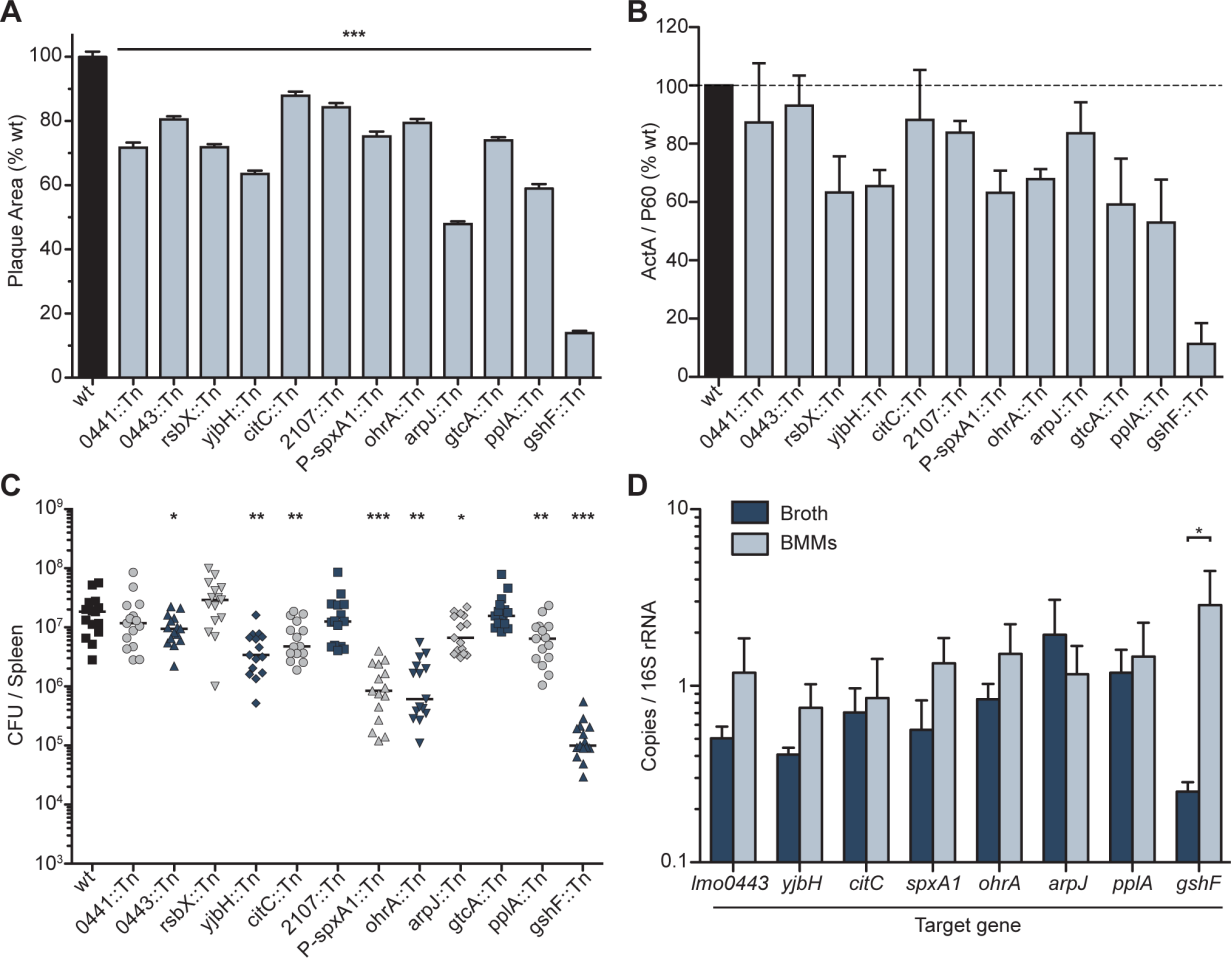
Characterization of mutants identified in the genetic selection. **(A)** Plaque area as a percentage of wild type. Data are the mean and error bars indicate the standard error of the mean (s.e.m.) for three independent experiments. *p* values were calculated using a heteroscedastic Student’s *t*-test and all strains are significantly different from wild type (*p* < 0.001). **(B)** Quantification of immunoblots of ActA and P60 during infection. ActA abundance was normalized to P60 abundance and measured as a percentage of wild type. Data are the mean ± s.e.m. of at least three independent experiments. **(C)** Female CD-1 mice were infected with 10^5^ colony forming units (CFU) of each mutant. Spleens were harvested 48 hours post-infection and CFU were quantified. The solid lines indicate the median, and data represent three pooled experiments totaling n = 15 mice per strain. *p* values were calculated using a heteroscedastic Student’s *t*-test * *p* < 0.05; ** *p* < 0.01; *** *p* < 0.001. **(D)** Gene expression of target genes measured by quantitative RT-PCR in wild type *L*. *monocytogenes* grown in broth compared to expression during infection of BMMs. Data are the mean ± s.e.m. of at least three independent experiments. *p* values were calculated using a heteroscedastic Student’s *t*-test * *p* < 0.05.

Transposon mutants in the suicide background were screened individually for survival in BMMs, narrowing the list to 300 mutants. Six transposon insertions were identified in *hly* and nine insertions in *prfA*, emphasizing that cytosolic access and PrfA are absolutely required for *actA* activation and subsequent *cre* expression. Saturation of the screen was further demonstrated after identification of 11 insertions in the *actA* promoter driving *cre* and 31 insertions in the *loxP* sites (which are each only 34 nucleotides). The remaining transposon mutations were transduced into a wild type background and analyzed in a plaque assay, a highly sensitive measure of cell-to-cell spread, which is completely dependent on *actA* expression [22]. Using a threshold of 85%, 12 mutants were identified that formed plaques significantly smaller than wild type in L2 murine fibroblasts (Fig 2A and Table 1). With one exception, the transposon insertions were in open reading frames and likely resulted in loss-of-function mutations. The transposon in the promoter of *lmo2191* (*spxA1*), a gene predicted to be essential in *L*. *monocytogenes* [23], resulted in a 10-fold decrease in *spxA1* expression when the bacteria were grown in broth, essentially resulting in a knock-down strain (S1 Fig). Attempts to make an in-frame deletion of *spxA1* using conventional methods were unsuccessful, consistent with a previous report [23].

**Table 1:** Genes identified in the forward genetic selection.

Gene^a^	Name	Function	Plaque Size in L2 cells (% wt ± SEM)	Plaque Size in TIB-73 cells (% wt ± SEM)
*lmo0441*		penicillin-binding protein	71.70 ± 1.60	75.6 ± 3.2
*lmo0443*		similar to *B*. *subtilis* LytR/TagU (LCP family protein)	78.97 ± 1.18	77.8 ± 5.6
*lmo0896*	*rsbX*	Indirect regulator of sigma B-dependent gene expression (serine phosphatase)	71.87 ± 0.94	69.6 ± 4.3
*lmo0964*	*yjbH*	thiol oxidoreductase	63.56 ± 0.98	53.8 ± 4.3
*lmo1566*	*citC*	isocitrate dehydrogenase	82.52 ± 1.25	107.5 ± 5.7
*lmo2107*		DeoR family transcriptional regulator	84.28 ± 1.36	93.6 ± 6.7
*P-lmo2191* ^b^	*spxA1*	ArsC family transcriptional regulator	75.22 ± 1.52	107.0 ± 4.4
*lmo2199*	*ohrA*	hypothetical protein (peroxiredoxin, OsmC/Ohr family)	79.44 ± 1.25	100.6 ± 5.9
*lmo2250*	*arpJ*	polar amino acid ABC transporter	47.90 ± 0.82	60.6 ± 2.0
*lmo2549*	*gtcA*	wall teichoic acid glycosylation protein	73.98 ± 0.94	83.6 ± 4.5
*lmo2637*	*pplA*	conserved lipoprotein	59.00 ± 1.39	77.9 ± 3.6
*lmo2770*	*gshF*	glutathione synthase	13.92 ± 0.72	28.4 ± 3.4

^a^ Gene loci based on *L*. *monocytogenes* EGD-e genome.

^b^ Transposon insertion in the predicted promoter of *lmo2191*.

As the goal of this selection was to identify mutations that affect ActA expression *in vivo*, we measured ActA abundance during infection of BMMs. Four hours post-infection, cells were lysed and ActA and the constitutively expressed P60 protein were analyzed by immunoblot. Nine strains were found to express less ActA than wild type after normalizing to P60 abundance (Fig 2B). The work-flow of this selection used *cre* expression from the *actA* promoter and plaque area as a criterion for inclusion in the core set of twelve mutants analyzed here. It was therefore unexpected that three mutants (*lmo0441*::*Tn*, *lmo0443*::*Tn*, and *citC*::*Tn*) did not display a defect in ActA abundance during intracellular growth. We hypothesize that these mutations may disrupt elements of bacterial physiology critical to appropriate Cre activity or normal growth.

### Virulence

The twelve mutants isolated by the genetic selection were identified based on *in vitro* assays for virulence. While these assays are correlated to *in vivo* outcomes, the importance of these genes to *L*. *monocytogenes* pathogenesis was confirmed in a murine model of infection. Intravenous infection of mice revealed that four of the mutants displayed no virulence defect (*lmo0441*::*Tn*, *rsbX*::*Tn*, *lmo2107*::*Tn*, and *gtcA*::*Tn*) while the remaining eight mutants were significantly attenuated (Fig 2C). It was surprising that four mutants exhibited impaired plaque formation yet were fully virulent; it is possible that these four mutants are impaired in other aspects of pathogenesis not reflected by changes in CFU during these infection conditions. To determine if the plaque defects in these mutants were due to cell-specific defects evident only in the L2 murine fibroblasts used for plaque assays, cell-to-cell spread defects were also analyzed in TIB-73 cells, a murine hepatocyte cell line (Table 1). We observed consistent phenotypes between the plaque defects in TIB-73 cells and L2 cells with the exception of *citC*::*Tn*, *P-spxA1*::*Tn*, and *ohrA*::*Tn*. However, these mutants were significantly attenuated during infection and thus it was unclear why they did not display a plaque defect in TIB-73 cells.

The specificity of the transposon insertion in seven of the eight attenuated strains was confirmed by expressing the disrupted gene *in trans* and complementing the plaque defect (S2 Fig). Attempts to complement the *pplA*::*Tn* plaque defect were unsuccessful. However, *pplA* mutants are difficult to complement and the mutant we identified exhibited phenotypes consistent with published *ΔpplA* defects [24]. Other reports have identified genes necessary for virulence of *L*. *monocytogenes* by comparing changes in gene expression *in vivo* [25–27]. In our analysis, only *gshF* was differentially transcribed between host cells and rich media (Fig 2D). It remains to be investigated if the activity of these genes is regulated post-transcriptionally in response to the host.

In this study we focused on the following genes that were required for *actA* expression and pathogenesis (Fig 1B). *yjbH* (*lmo0964*) encodes a putative thioredoxin similar to YjbH in *Bacillus subtilis* (57% amino acid similarity) [28]. A transposon in *L*. *monocytogenes yjbH* was previously identified in a screen for mutants defective in LLO production *in vitro* and was found to be attenuated in a competitive infection model [29]. *spxA1* (*lmo2191*) encodes an ArsC family transcriptional regulator similar to the disulfide stress regulator Spx conserved in Firmicutes (83% amino acid identity to *B*. *subtilis* Spx) [30]. The difference in nomenclature is due to the presence of a paralogous gene in *L*. *monocytogenes* (*lmo2426* or *spxA2*) that is 59% identical to *B*. *subtilis* Spx while *B*. *subtilis* encodes only a single *spx*. In *B*. *subtilis* and *Staphylococcus aureus* YjbH post-translationally regulates Spx [28,31], although it is not known if this function is conserved in *L*. *monocytogenes*. *lmo2199* encodes a hypothetical protein with a peroxiredoxin domain and is part of the organic hydroperoxide resistance (Ohr) protein subfamily. It is co-transcribed with *lmo2200*, encoding a MarR family transcriptional regulator which was not required for virulence, suggesting that Lmo2200 may act as a transcriptional repressor [26]. In *B*. *subtilis* homologs of Lmo2199 and Lmo2200 are named OhrA (63% amino acid similarity) and OhrR (68%), respectively, and we have adopted this nomenclature for consistency [32]. *arpJ* (*lmo2250*) encodes a predicted amino acid ABC transporter permease that was originally identified in a screen for genes with increased intracellular expression [25]. However, the data presented here did not show an increase in *arpJ* expression during infection of BMMs. This may be explained by the different growth media and cell types used in the two studies. It is also possible that *arpJ* is autoregulated, as the previous study analyzed *arpJ* expression in an *arpJ* transposon mutant. *pplA* (*lmo2637*) encodes a lipoprotein whose secretion is increased in a PrfA* mutant [33]. The signal sequence of this lipoprotein is processed into a secreted peptide, which is required for vacuolar escape from non-phagocytic cells [24]. Finally, *gshF* (*lmo2770*) encodes the only glutathione synthase in *L*. *monocytogenes* [34]. Glutathione has been demonstrated to be an allosteric activator of PrfA and therefore *gshF* mutants are severely attenuated *in vivo* due to insufficient virulence gene expression [17].

### *In vivo* suppressor analysis to dissect PrfA abundance versus activation

**Fig 3:**
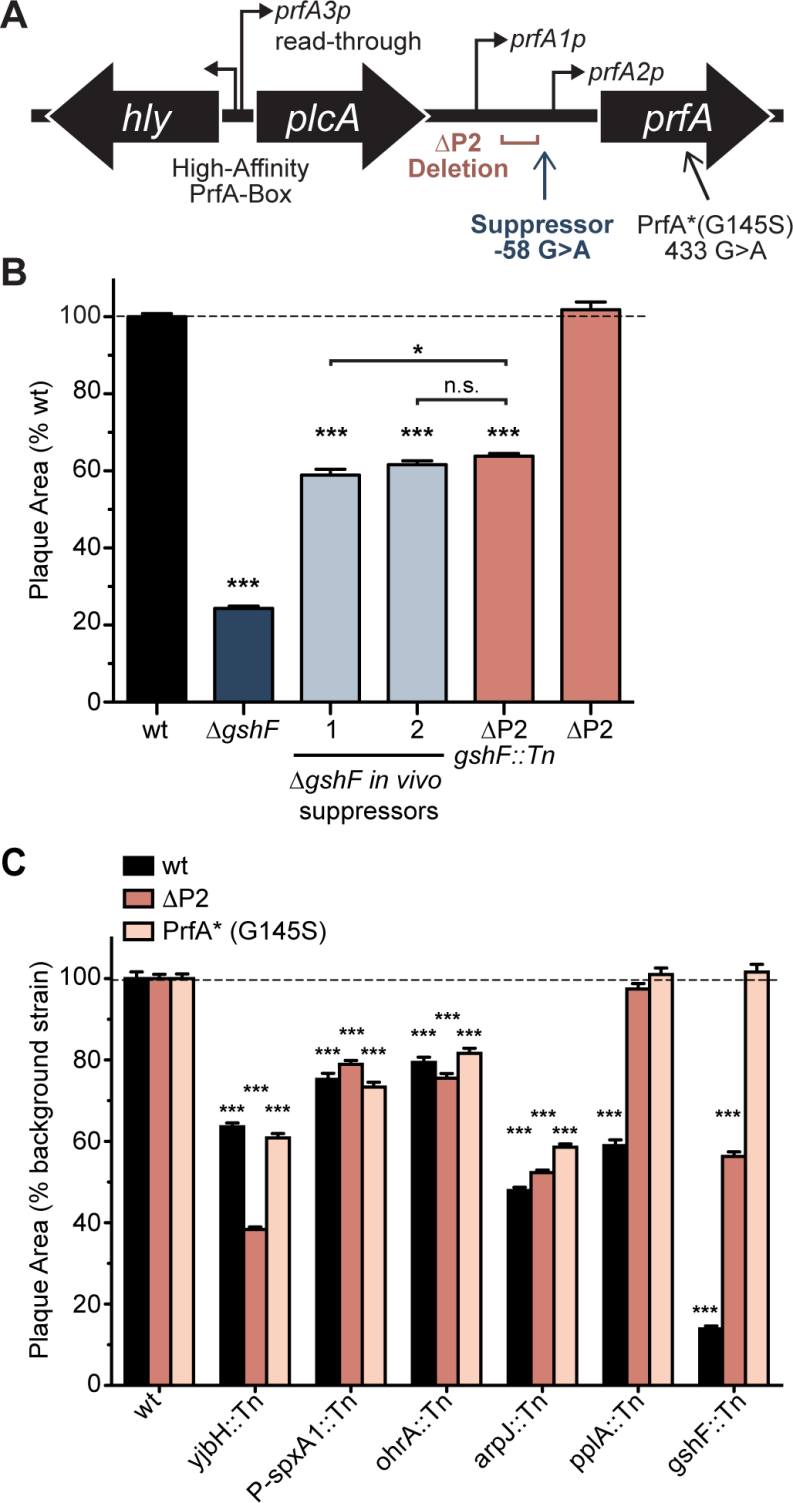
*In vivo* suppressor analysis. **(A)** Schematic of the *prfA* region. Thin black arrows represent transcription start sites [11]**. (B)** Plaque area as a percentage of wild type. **(C)** Plaque area as a percentage of the indicated background strain. For panels B and C: data are the mean ± s.e.m. of at least three independent experiments and *p* values were calculated using a heteroscedastic Student’s *t*-test *** *p* < 0.001; n.s. *p* > 0.05.

Given the role of glutathione in activating PrfA, we hypothesized that suppressor mutations of Δ*gshF* might illuminate alternative pathways for PrfA activation, potentially involving other genes identified. Accordingly, we screened for mutations that increased the virulence of a Δ*gshF* mutant. Mice were serially infected with a high-inoculum of Δ*gshF*, livers were harvested at 72 hours p.i., homogenized, and diluted to inoculate naive mice. After four successive infections bacteria isolated from infected livers were analyzed by plaque assay. This approach previously identified a mutation in *prfA* that constitutively activates the protein (G145S), known as PrfA*, completely bypassing the requirement for glutathione during infection [17]. The Δ*gshF* PrfA* suppressor forms 100% plaque; therefore, for these experiments we selected bacteria that formed intermediate-sized plaques, which were then subjected to genome sequencing. Two suppressor mutants were isolated and found to encode a G>A mutation 58 nucleotides 5’ of the *prfA* start codon (Fig 3A). This mutation lies within a previously identified site of negative regulation of *prfA*, the so-called “P2 promoter” (*prfA2p*, Fig 3A) and deletion of the -35 region of this promoter (ΔP2 mutant) results in a 10-20-fold up-regulation of the *prfA1p*-dependent *prfA* transcript [11]. We hypothesized that the *prfA* -58 G>A mutation also inactivated the P2 promoter and resulted in greater PrfA abundance. Indeed, the ΔP2 *gshF*::*Tn* double mutant and the Δ*gshF prfA* -58 G>A suppressor mutants all formed plaques approximately 60% the size of wild type (Fig 3B). These results did not directly implicate any of the other genes identified in our genetic selection, however these findings did highlight the impact of both PrfA abundance and activation during infection.

PrfA expression is controlled by a feed-forward loop in which activated PrfA drives its own transcription [12]. Strains expressing ΔP2 or PrfA* decouple PrfA abundance and activation whereby ΔP2 increases PrfA abundance but still relies on glutathione for PrfA activation; PrfA* increases both the amount and activity of PrfA, independent of glutathione. We next sought to determine if the other mutants identified in the screen affected PrfA abundance or activation by transducing each into *L*. *monocytogenes* ΔP2 and PrfA* backgrounds and measuring the plaque size in each background (Fig 3C). Based on these analyses, mutants fell into three categories. The first category (*yjbH*::*Tn*, *P-spxA1*::*Tn*, *ohrA*::*Tn*, and *arpJ*::*Tn*) was unaffected by alterations in PrfA expression or activity, indicating that these genes were required down-stream of PrfA. In the second category was *gshF*::*Tn*, which was partially rescued by ΔP2 and completely rescued by PrfA*, consistent with the demonstrated role for glutathione as the allosteric activator of PrfA. The third category describes *pplA*::*Tn*, which formed 100% plaques in both the ΔP2 and PrfA* backgrounds. These data suggested that the *pplA* mutant was capable of activating PrfA (because it was rescued by ΔP2) but was deficient in expression of PrfA-dependent genes required early during infection before cytosolic access and glutathione-mediated activation of PrfA.

### Vacuolar escape and cytosolic growth

**Fig 4:**
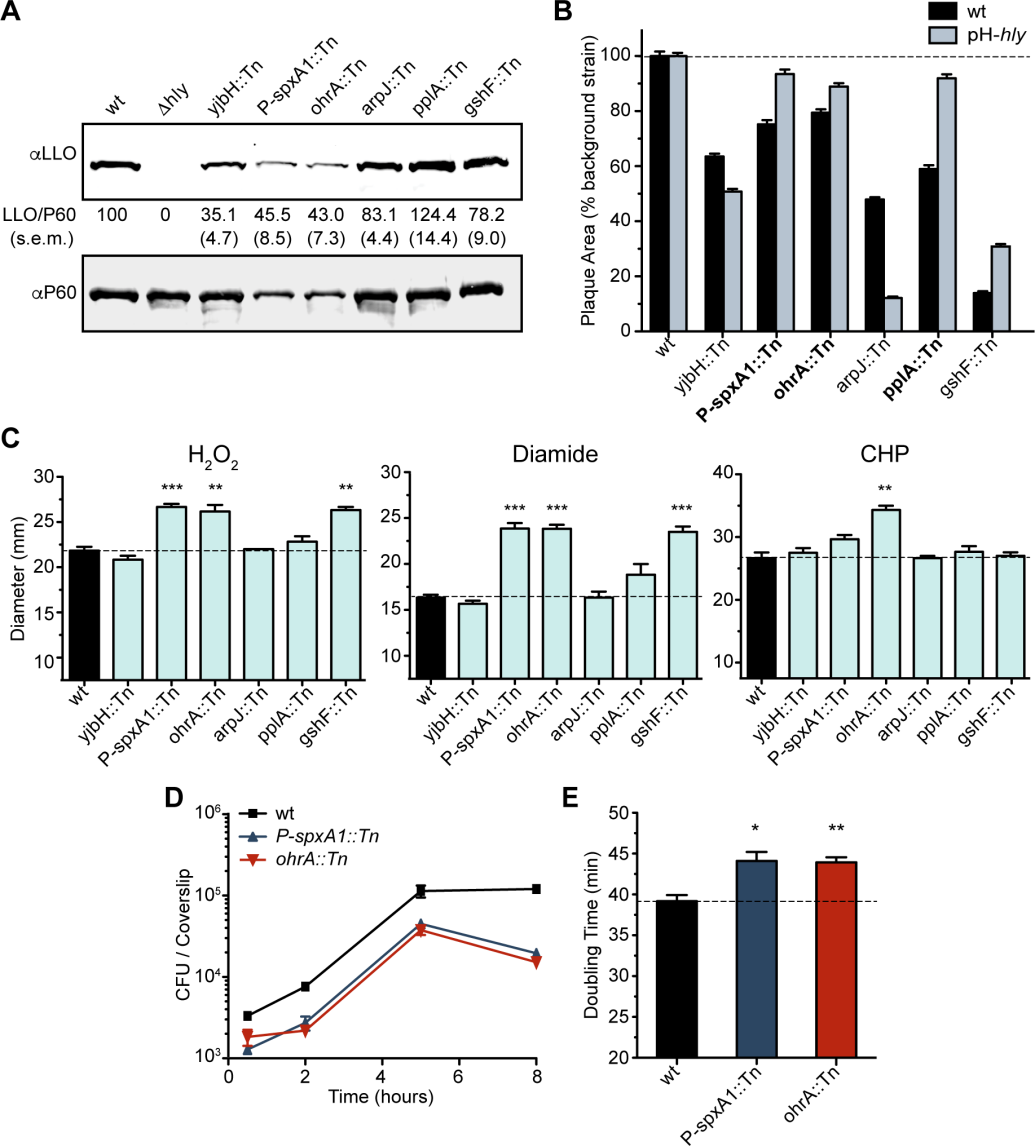
Mutants impaired for vacuolar escape. **(A)** Representative immunoblots of the secreted proteins LLO and P60. LLO abundance was normalized to P60 abundance and measured as a percentage of wild type. Data are the mean ± s.e.m. of at least three independent experiments. **(B)** Plaque area as a percentage of the indicated background strain. The mutants that were rescued by *pH-hly* are in bold. Data are the mean ± s.e.m. of at least three independent experiments. **(C)** Sensitivity of mutants to hydrogen peroxide (5% v/v), diamide (1 M), and cumene hydroperoxide (CHP, 80% v/v) as measured by growth inhibition in a disk diffusion assay. Dotted line corresponds to the wild type diameter for comparison. The disks were 7.5 mm in diameter. Data are the mean ± s.e.m. of at least three independent experiments and *p* values were calculated using a heteroscedastic Student’s *t*-test ** *p* < 0.01; *** *p* < 0.001. **(D)** BMM growth curve. Data indicate the mean and s.e.m. of three technical replicates and are representative of three independent experiments. **(E)** Log phase doubling time of mutants grown shaking in broth. Data are the mean ± s.e.m. of at least three independent experiments. *p* values were calculated using a heteroscedastic Student’s *t*-test * *p* < 0.05; ** *p* < 0.01.

A principle difference between early and late PrfA-dependent genes is that expression of early genes are less dependent on PrfA activation by glutathione [35]. The two early genes are *hly* (encoding LLO) and *plcA*, which share a high-affinity PrfA-box and are transcribed by unactivated PrfA [35,36]. The ΔP2 mutation results in increased transcription of early genes but does not affect late gene expression, whereas PrfA* increases transcription of both early and late genes. We hypothesized that strains rescued by ΔP2 are specifically deficient in early gene expression. Accordingly, we analyzed early gene expression (LLO production) in broth for each mutant. Several of the mutants were found to secrete less LLO than wild type (Fig 4A). To determine if the defect in LLO production led to impaired phagosomal escape and thus a plaque defect, these mutations were transduced into a *Δhly* mutant over-expressing *hly* from a constitutive HyPer promoter (*pH*-*hly* strain) [37,38]. In this background, efficiency of vacuolar escape should be equivalent in all strains, and indeed, equal LLO secretion was confirmed in broth. Constitutive expression of *hly* rescued the plaque defects of three mutants: *P-spxA1*::*Tn*, *ohrA*::*Tn*, and *pplA*::*Tn* (Fig 4B). Interestingly, there was discordance between LLO production in broth and the defect in plaque formation one might predict from an LLO deficiency. For this reason, measuring LLO production in broth may be revealing aspects of bacterial physiology unrelated to LLO production *in vivo*.

The above results suggested that mutations in *P-spxA1*, *ohrA*, and *pplA* resulted in aberrant LLO secretion and/or that these mutants were unable to survive in the harsh environment of the vacuole. Constitutive expression of *hly* would likely overcome either defect. We attempted to segregate these two possibilities by analyzing sensitivity to vacuolar conditions, including reactive oxygen species which *L*. *monocytogenes* must adapt to in order to survive [39,40]. The response of each mutant to peroxide, disulfide stress, and organic hydroperoxide was analyzed by measuring their sensitivity to hydrogen peroxide (H_2_O_2_), diamide, and cumene hydroperoxide (CHP), respectively. Knock-down of *spxA1* and disruption of *ohrA* or *gshF* significantly increased the sensitivity of *L*. *monocytogenes* to both peroxide and disulfide stress (Fig 4C). In accordance with its annotation and the published role of *ohrA* in *B*. *subtilis* [32], the *ohrA*::*Tn* mutant was significantly more susceptible to CHP (Fig 4C). As these results suggested a role for redox control of virulence genes, we tested the hypothesis that host reactive oxygen or nitrogen species may be sensed by the bacteria during infection to activate *actA*. However, growth of the suicide mutant was not rescued in BMMs lacking inducible nitric oxide synthase (*NOS2* ^*-/-*^) or NADPH oxidase (*NOX2* ^*-/-*^) (S3 Fig). Therefore, *L*. *monocytogenes* may activate virulence genes in response to multiple redundant host cues or depend on yet unidentified host pathways.

Constitutive production of *hly* restored the majority of the plaque defect for *P-spxA1*::*Tn* and *ohrA*::*Tn*, however, it did not restore the plaque to 100% of the parent strain (Fig 4B). We hypothesized that these mutants might also be impaired in the ability to grow in the host cytosol, independently from virulence gene expression. All of the mutants identified in the screen grew similarly to wild type in BMMs with the exception of *P-spxA1*::*Tn* and *ohrA*::*Tn* (Fig 4D). In fact, *P-spxA1*::*Tn* and *ohrA*::*Tn* were also the only mutants that exhibited growth defects in rich media (Fig 4E). These pleiotropic growth defects and sensitivity to redox stress are likely why *pH*-*hly* was only partially able to complement the plaque defect of these mutants (Fig 4B).

### YjbH is necessary for ActA translation

**Fig 5:**
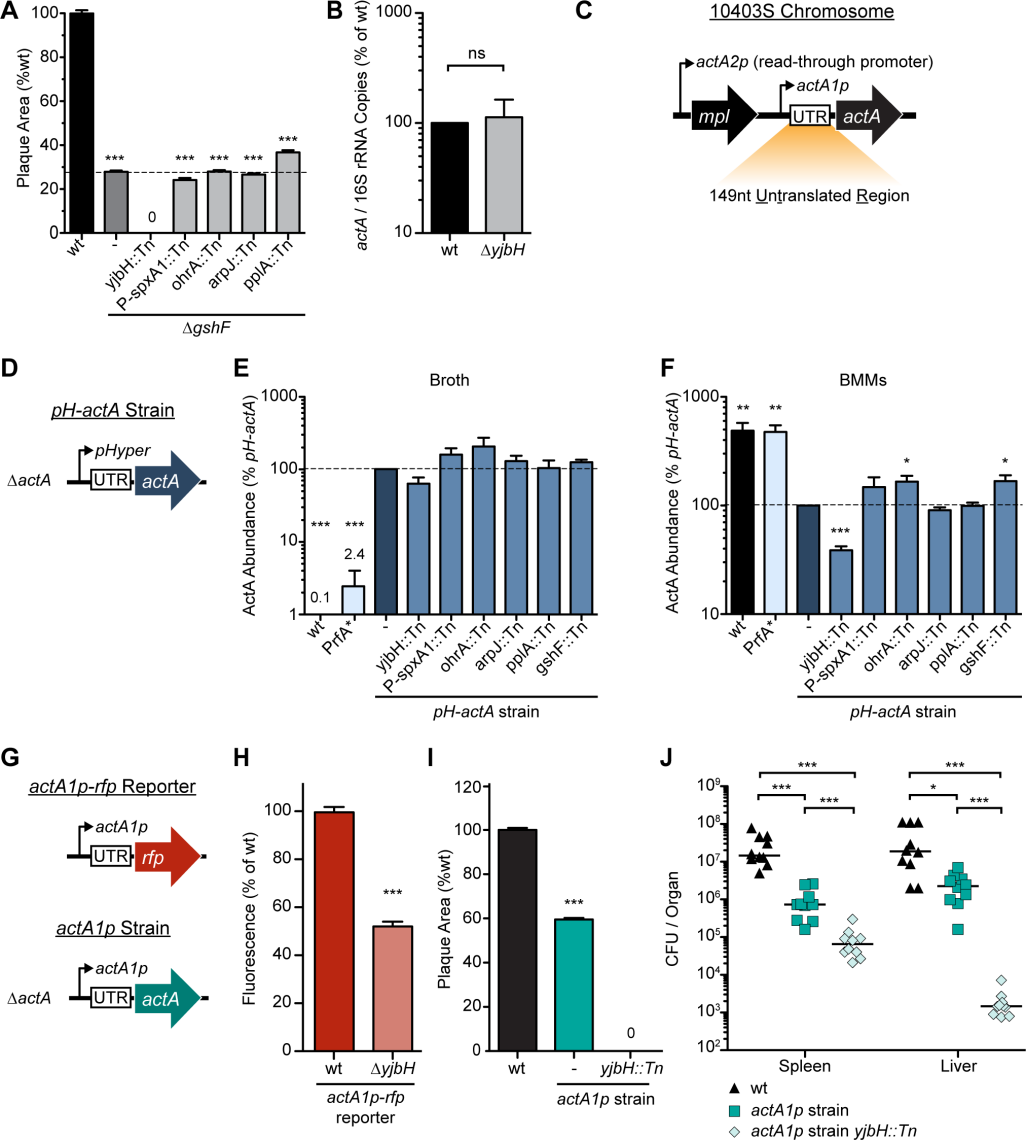
Post-transcriptional activation of ActA. **(A)** Plaque area as a percentage of wild type. **(B)** qPCR of *L*. *monocytogenes* transcripts during BMM infection. For panels A and B, data are the mean ± s.e.m. of at least three independent experiments. **(C)** Schematic of the *actA* region in the chromosome. Thin black arrows represent predicted transcription start sites [15]. **(D)** Schematic of the constitutive *pH-actA* strain. **(E)** *In vitro* abundance of ActA normalized to P60 was measured by immunoblot and plotted as a percentage of the *pH-actA* strain during broth growth. Data are the mean ± s.e.m. of at least three independent experiments. **(F)** Abundance of ActA normalized to P60 was measured during BMM infection by immunoblot and plotted as a percentage of the *pH-actA* strain four hours post-infection of BMMs. Data are the mean ± s.e.m. of at least three independent experiments. **(G)** Schematic of the *actA1p-rfp* reporter strain and the *actA1p* strain. **(H)** RFP fluorescence six hours post-infection of BMMs with the *actA1p-rfp* reporter strains. Data are the mean ± s.e.m. of at least three independent experiments. **(I)** Plaque area as a percentage of wild type. Data are the mean ± s.e.m. of at least three independent experiments. **(J)** Female CD-1 mice were infected with 10^5^ CFU of each mutant. Spleens and livers were harvested 48 hours post-infection and CFU were quantified. The solid lines indicate the median, and data represent two pooled experiments totaling n = 10 mice per strain. In all panels, *p* values were calculated using a heteroscedastic Student’s *t*-test * *p* < 0.05; ** *p* < 0.01; *** *p* < 0.001; ns (not significant) *p* > 0.05.

Previous work clearly demonstrated that glutathione was essential for transcriptional activation of virulence genes [17]. In order to assess which factors might be independent of glutathione-dependent transcriptional activation, we combined each transposon with an in-frame *ΔgshF* mutation. The only mutation not epistatic to *gshF* was *yjbH*::*Tn*, which produced an additive plaque defect (Fig 5A). Further, *yjbH*::*Tn* was not rescued by constitutive activation of *hly* (Fig 4B) or *prfA* (Fig 3C). Together, these data suggested that *yjbH* was required for *actA* expression post-transcriptionally. Indeed, transcript levels of *actA* were identical in BMMs infected with wild type or the Δ*yjbH* mutant (Fig 5B). It is intriguing that *arpJ*::*Tn* was epistatic to *gshF*, yet not rescued by constitutive activation of PrfA, indicating that *arpJ* may contribute to glutathione-dependent transcriptional activation of *actA* through an unknown mechanism.

The *actA* gene is preceded by 149 nucleotides of untranslated mRNA (Fig 5C) which is important for sufficient ActA expression [21]. A strain was constructed in which ActA was expressed independent of PrfA by expressing the entire actA transcript (including the 5’ UTR) under the control of the constitutive HyPer promoter in a strain deleted for *actA* (*pH*-*actA* Strain, Fig 5D). ActA protein abundance was then analyzed by immunoblot. In this background, ActA abundance was equivalent among all strains when the bacteria were grown in broth (Fig 5E). However, during infection of BMMs, disruption of *yjbH* resulted in significant impairment in ActA abundance (Fig 5F), indicating a failure to translationally activate actA. Given that disrupting *yjbH* rescued the death of the suicide strain in which *cre* was expressed under *actA1p* and the 5' UTR, these data indicate a genetic interaction between *yjbH* and the 5’ UTR of *actA*. To further support this genetic interaction we engineered a fluorescent strain of *L*. *monocytogenes* in which *rfp* was expressed under the *actA1p* promoter and 5' UTR (*actA1p*-*rfp*, Fig 5G). During infection of BMMs the Δ*yjbH actA1p*-*rfp* strain exhibited significantly less fluorescence than wild type *actA1p*-*rfp* (Fig 5H). Unfortunately, we were unable to interrogate the effect of a *yjbH* mutation on ActA abundance in the absence of its 5’ UTR due to an inability to detect ActA when the 5’ UTR was deleted, consistent with this region being critical for ActA expression [21].

A drawback to *pH*-*actA* is that although ActA is over-expressed in broth, this strain still elaborates much less ActA *in vivo* and fails to form a plaque (Fig 5E and 5F). To analyze the role of translational activation during infection, the *actA* gene and 5’ UTR were moved to a neutral locus within the *L*. *monocytogenes* chromosome [41]. In this strain, *actA* was expressed only from the PrfA-dependent *actA1p* proximal promoter, eliminating read-through transcription from the distal *actA2p* promoter (Fig 5C). This strain was called *actA1p* and was only mildly impaired in plaque formation and virulence (Fig 5I and 5J). However, *actA1p yjbH*::*Tn* was unable to form a plaque (Fig 5I). The importance of actA translational activation was further underscored by a 3-log defect for *actA1p yjbH*::*Tn* in the livers of infected mice (Fig 5J). These data revealed a critical role for *yjbH* in actA activation that was less apparent in the wild type background due to redundant PrfA-dependent promoters.

## Discussion

**Fig 6:**
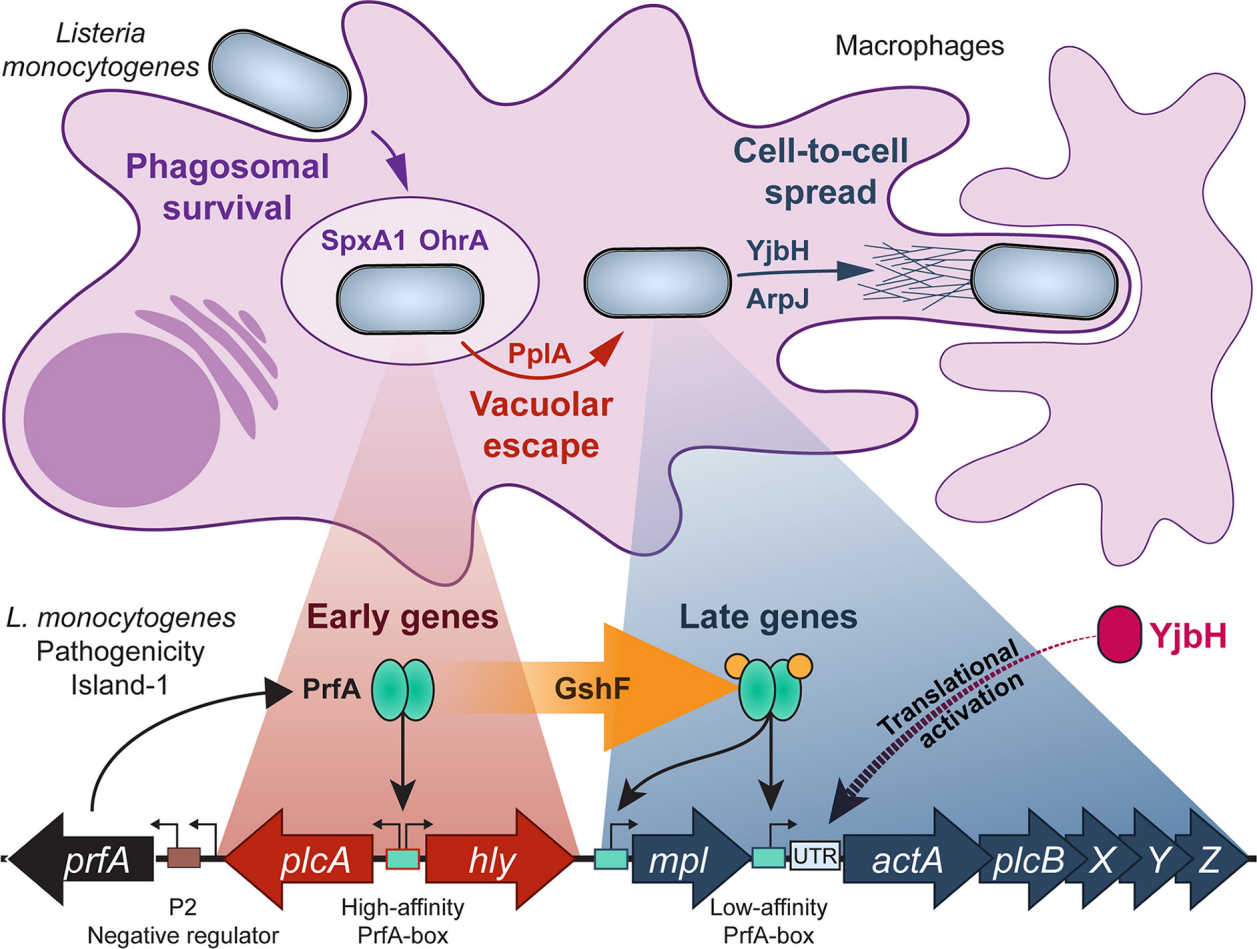
Model of genes identified in this genetic selection and where in the *L*. *monocytogenes* life cycle they are required. Once phagocytosed by a host macrophage, *L*. *monocytogenes* (light blue rods) requires the gene products of *spxA1* and *ohrA* to survive in the phagosome. By a mechanism that is not yet understood, PplA is required for vacuolar escape in non-phagocytic cells. YjbH and ArpJ are then required for cell-to-cell spread. The *L*. *monocytogenes* Pathogenicity Island-1 is pictured below. Early genes (depicted in red) are those with high-affinity PrfA boxes that do not require active PrfA (teal) for transcription. Late genes (depicted in blue) are those with relatively low-affinity PrfA boxes that require activated PrfA to be transcribed and these are required later during infection, in the host cytosol. The transition from unactivated to activated PrfA requires glutathione (orange circles), which is synthesized by GshF. YjbH (magenta) is then required for translational activation of actA, although the mechanism is not yet understood. See text for more details, model is not drawn to scale.

In this study, rather than search for novel virulence factors or genes up-regulated *in vivo*, we screened for genes required for activation of an essential determinant of *L*. *monocytogenes* pathogenesis (ActA) that is up-regulated over 200-fold during intracellular growth. Mutants identified in the genetic selection fell into three broad categories: (1) those that failed to reach the cytosolic compartment; (2) mutants that entered the cytosol, but failed to activate the master virulence transcriptional regulator PrfA; and (3) mutants that entered the cytosol and activated transcription of *actA*, but failed to synthesize it (Fig 6). This approach highlighted how expression of virulence factors is spatially and temporally compartmentalized via regulation of transcription and translation during infection. One of the most striking findings of this study was that the majority of genes identified in the selection encode proteins predicted to control bacterial redox regulation, suggesting that redox changes represent one of the biological cues sensed by *L*. *monocytogenes* to regulate its virulence program. Redox stress during infection can arise from endogenous by-products of bacterial metabolism and exogenously derived factors generated by the host. However, it remains to be discovered whether the redox stress that may trigger virulence gene expression is produced by the host, the bacteria, or both.

YjbH, Spx, OhrA, and GshF have defined roles in maintaining redox homeostasis in the presence of disulfide and organic peroxide stresses in Firmicutes. In *B*. *subtilis* OhrA is a peroxiredoxin required during organic hydroperoxide stress [32]. In *S*. *aureus* and *B*. *subtilis* YjbH interacts with Spx to regulate the abundance and activity of Spx [28,31]. Specifically, YjbH-bound Spx is recognized by the ClpXP protease and is degraded so that Spx concentrations are low under steady-state conditions [42,43]. During disulfide stress the YjbH:Spx interaction is disrupted by intramolecular disulfide bonds in both proteins that result in reduced proteolysis of Spx. *B*. *subtilis* Spx represses transcription of 176 genes and activates transcription of 106 genes [44], the majority of which are required to adapt to redox stress, including genes for production of the low-molecular weight (LMW) thiol utilized by *B*. *subtilis*, bacillithiol [45]. *L*. *monocytogenes spxA1* cannot be deleted and its regulon has not yet been characterized [23]. Similarly, in *Streptococcus pneumoniae* simultaneous deletion of both *spxA1* and *spxA2* paralogues is lethal [46], supporting the notion that the Spx regulon(s) may contain essential genes in some Firmicutes.

Mutants exhibiting the most severe virulence phenotypes contained insertions in *gshF*, which encodes the sole *L*. *monocytogenes* glutathione synthase [34]. Glutathione is a tripeptide LMW thiol antioxidant present at millimolar concentrations that contributes to maintaining a reducing environment in both bacterial and host cells [47]. Not surprisingly, *L*. *monocytogenes* Δ*gshF* mutants are more sensitive to redox stressors such as hydrogen peroxide and diamide and are 200-fold less virulent in mice, indicating that bacterially-derived glutathione is essential for pathogenesis [17]. However, Δ*gshF* mutants are fully virulent in *L*. *monocytogenes* harboring prfA* mutations that lock PrfA in its constitutively active conformation. Therefore, the primary role of GshF-derived glutathione during infection is to activate virulence gene expression via PrfA activation, although we cannot rule out a contribution of imported host-derived glutathione [17]. Indeed, host-derived glutathione activates virulence gene expression in *Burkholderia pseudomallei* [48]. In the case of *L*. *monocytogenes*, *gshF* is transcriptionally up-regulated 10-fold during intracellular growth, suggesting the existence of an unidentified cue, likely redox-related, that stimulates glutathione production.

The identification of many redox-related bacterial factors in this genetic selection led to our working model that specific redox changes during infection are sensed by the bacteria as a mechanism to identify their intracellular location and activate virulence genes appropriately. Redox stress during infection could arise from host-derived antimicrobial factors. For example, the host generates antibacterial factors that assault invading pathogens with redox stresses, including: reactive oxygen species (ROS), reactive electrophilic species (RES) such as methylglyoxal, and reactive nitrogen species (RNS) such as nitric oxide and peroxynitrite [40,49,50]. Interestingly, these redox stresses from the host are spatially compartmentalized. RNS and ROS are produced in the phagosome and once in the host cytosol, *L*. *monocytogenes* is confronted with RES and mitochondrial-derived ROS [40,51]. It is possible that the bacterial response to the redox stressors is also compartmentalized, requiring specific factors in the vacuole (such as *spxA1* and *ohrA*) and host cytosol (such as *yjbH*).

Eliminating host nitric oxide synthase (NOS2) or NADPH oxidase did not rescue growth of the suicide mutant (S3 Fig). NOS2-generated nitric oxide is required for efficient *L*. *monocytogenes* cell-to-cell spread during infection, although this is due to the nitric oxide-mediated delay of phagolysosome maturation and not a direct effect on the bacteria [52]. Together, these data suggest that a combination of host factors are likely required to activate *actA* during infection.

Alternatively, the source of redox stress may come from bacterial metabolism via ROS generated from incomplete reduction of oxygen during aerobic respiration [53]. Carbon source and phosphate abundance also affect the production of ROS and methylglyoxal [54,55]. PrfA activity has been demonstrated to be sensitive to available carbon sources [2]. Growth on plant-derived beta-glucoside sugars in the environment, such as cellobiose, represses PrfA activation, whereas growth on host-derived sugars such as glucose-1-phosphate stimulates PrfA-dependent gene expression [9,56,57]. Therefore, entry of *L*. *monocytogenes* into the host cytosol results in a remodeling of carbon metabolism that may be linked to virulence gene regulation. Glycerol is the principle carbon source used by *L*. *monocytogenes* intracellularly and growth on glycerol is a well-described stimulant of methylglyoxal production [58–61]. In *B*. *subtilis*, methylglyoxal stress stimulates the Spx regulon and production of bacillithiol, a low molecular weight thiol used by *B*. *subtilis* to detoxify methylglyoxal [62]. Thus, the 10-fold increase in *gshF* transcript levels in *L*. *monocytogenes* may correspond to increased methylglyoxal production during infection, which would further link metabolism of an alternative carbon source to virulence. Coupling of metabolism to virulence gene regulation may allow the system to remain OFF in the environment while remaining poised to turn ON upon entering a host. Considering our finding of multiple redox factors that are required for proper virulence gene expression, we speculate that changes in carbon metabolism could alter the endogenous levels of ROS and RES produced, thus affecting PrfA activation and leading to the “sugar-mediated repression” observed previously [9].

Appropriate up-regulation of actA at the translational level is understood to require its 5’ UTR, although the mechanism remains unknown [21]. The data reported here further emphasize the sensitivity of actA translation to the environment in which *L*. *monocytogenes* is growing. In broth, the PrfA* strain elaborated 2.4% the amount of ActA protein as compared to constitutively expressed *actA* (Fig 5E), and increased 200-fold during infection (Fig 5F), despite the fact that transcript levels of actA are equivalent in both growth conditions [17]. These data emphasize the importance of the translational control of this virulence factor. Importantly, *yjbH* was required for the increased abundance of ActA protein during infection. In wild type *L*. *monocytogenes*, multiple PrfA-dependent promoters may compensate for loss of translational activation; however, when *actA* was isolated under its most proximal promoter, disruption of *yjbH* resulted in an attenuation of over 3-logs in the livers of infected animals (Fig 5J). It seems unlikely that the thioredoxin YjbH activates translation of actA via direct binding to the 5’ UTR. However, YjbH may indirectly activate translation via interaction with another factor(s) or modulation of a small-molecule signal produced by the host.

PrfA-dependent transcription and activation are regulated redundantly at multiple levels, including: a temperature-sensitive riboswitch [63], allosteric activation by glutathione [17], multiple read-through transcripts [10,64], positive and negative promoter elements [11,65], and yet to be fully characterized translational control. The complexity of *actA* activation is likely the result of selective pressure to respond appropriately to host-derived cues. This study investigated the virulence defects associated with failure to up-regulate virulence genes; however, over-production or inappropriate regulation of virulence factors extracellularly also results in a competitive disadvantage for *L*. *monocytogenes* [19,20]. How *L*. *monocytogenes* and other intracellular pathogens regulate virulence gene expression is central to understanding their pathogenesis. Results reported here suggest that redox cues are a mechanism by which intracellular pathogens recognize the host and represents an exciting new area of further investigation.

## Methods

### Ethics statement

This study was carried out in strict accordance with the recommendations in the Guide for the Care and Use of Laboratory Animals of the National Institutes of Health. All protocols were reviewed and approved by the Animal Care and Use Committee at the University of California, Berkeley (AUP-2016-05-8811).

### Bacterial culture and strains

All *L*. *monocytogenes* strains are a derivative of wild type 10403S [67,68] and were cultivated in Brain Heart Infusion (BHI, Difco), shaking at 37°C unless otherwise stated. All *E*. *coli* strains were cultivated shaking in LB (Miller) at 37°C. Antibiotics (purchased from Sigma) were used at the following concentrations: carbenicillin (100 μg/mL), streptomycin (200 μg/mL), chloramphenicol (7.5 μg/mL for *L*. *monocytogenes* and 10 μg/mL for *E*. *coli*), erythromycin (1 μg/mL), and tetracycline (2 μg/mL). All *E*. *coli* strains are listed in Table 2 and all *L*. *monocytogenes* strains are listed in Table 3. Bacterial broth growth curves were performed as previously described [69]. The suicide strain was a gift from Peter Lauer and Bill Hanson (Aduro Biotech); details of its construction are reported elsewhere [17]. Briefly, *loxP* sites were inserted on either side of the origin of replication by allelic exchange into a *ΔactAΔinlB* strain of *L monocytogenes*. A transcriptional fusion of *cre* with *actA* that included the *actA1p* promoter, 5’ UTR, and ribosomal binding site of *actA*, was inserted adjacent to a *loxP* site.

**Table 2:** *Escherichia coli* strains.

Strain	Description	Reference
XL1-Blue	For vector construction	Stratagene
SM10	For trans-conjugation	[70]
DP-E6333	XL1-Blue pPL2t	[71]
DP-E6415	SM10 pPL2t.P_hyper_-*hly*	[72]
DP-E6416	SM10 pPL2t.P_hyper_-*actA*	This study
DP-E6475	SM10 pPL2.*yjbH*.His	This study
DP-E6476	XL1 pPL2.*spxA1*.His	This study
DP-E6477	SM10 pPL2t.*ohrRA*(*LMRG_01632-LMRG_01633)*	This study
DP-E6478	SM10 pPL2t.*arpJ* (*LMRG_01581-LMRG_01580*)	This study
DP-E6479	SM10 pPL2.*gshF*.His	[17]
DP-E6510	SM10 pPL2.*actA1p-TagRFP* (*actA1p-rfp* reporter)	[73]

**Table 3:** *Listeria monocytogenes* strains.

Strain	Description	Reference
10403S	wt	[68]
DP-L6186	‘suicide strain’ (BH-3410)	[17]
DP-L6419	*lmo0441*::*Tn*	This study
DP-L6420	*lmo0443*::*Tn*	This study
DP-L6421	*rsbX*::*Tn*, (*lmo0896*)	This study
DP-L6422	*yjbH*::*Tn*, (*lmo0964*)	This study
DP-L6423	*citC*::*Tn*, (*lmo1566*)	This study
DP-L6424	*lmo2107*::*Tn*	This study
DP-L6425	*P-spxA1*::*Tn*, (*lmo2191*)	This study
DP-L6426	*ohrA*::*Tn (lmo2199)*	This study
DP-L6427	*arpJ*::*Tn*, (*lmo2250*)	This study
DP-L6428	*gtcA*::*Tn*, (*lmo2549*)	This study
DP-L6429	*pplA*::*Tn*, (*lmo2637*)	This study
DP-L6430	*gshF*::*Tn*, (*lmo2770*)	This study
DP-L6188	*ΔgshF*	[17]
DP-L1866	Δ*prfA2p* -35 (*Δ*P2 strain)	[11]
DP-L5451	PrfA* (G145S)	[74]
DP-L6431	DP-L1866 + *yjbH*::*Tn*	This study
DP-L6432	DP-L1866 + *P-spxA1*::*Tn*	This study
DP-L6433	DP-L1866 + *ohrA*::*Tn*	This study
DP-L6434	DP-L1866 + *arpJ*::*Tn*	This study
DP-L6435	DP-L1866 + *pplA*::*Tn*	This study
DP-L6436	DP-L1866 + *gshF*::*Tn*	This study
DP-L6437	PrfA* *yjbH*::*Tn*	This study
DP-L6438	PrfA* *P-spxA1*::*Tn*	This study
DP-L6439	PrfA* *ohrA*::*Tn*	This study
DP-L6440	PrfA* *arpJ*::*Tn*	This study
DP-L6441	PrfA* *pplA*::*Tn*	This study
DP-L6191	PrfA* *gshF*::*Tn*	This study
DP-L4511	Δ*hly* pPL2.P_hyper_-*hly* (*pH-hly* strain)	[37]
DP-L6442	DP-L4511 + *yjbH*::*Tn*	This study
DP-L6443	DP-L4511 + *P-spxA1*::*Tn*	This study
DP-L6444	DP-L4511 + *ohrA*::*Tn*	This study
DP-L6445	DP-L4511 + *arpJ*::*Tn*	This study
DP-L6446	DP-L4511 + *pplA*::*Tn*	This study
DP-L6447	DP-L4511 + *gshF*::*Tn*	This study
DP-L6448	Δ*gshF yjbH*::*Tn*	This study
DP-L6449	Δ*gshF P-spxA1*::*Tn*	This study
DP-L6450	Δ*gshF ohrA*::*Tn*	This study
DP-L6451	Δ*gshF arpJ*::*Tn*	This study
DP-L6452	Δ*gshF pplA*::*Tn*	This study
DP-L6418	Δ*actA* pPL2t.P_hyper_-*actA* (*pH-actA* strain)	This study
DP-L6453	DP-L6418 + *yjbH*::*Tn*	This study
DP-L6454	DP-L6418 + *P-spxA1*::*Tn*	This study
DP-L6455	DP-L6418 + *ohrA*::*Tn*	This study
DP-L6456	DP-L6418 + *arpJ*::*Tn*	This study
DP-L6457	DP-L6418 + *pplA*::*Tn*	This study
DP-L6458	DP-L6418 + *gshF*::*Tn*	This study
DP-L4077	Δ*actA* pPL1.*actA1p-actA* (*actA1p* strain)	[41]
DP-L6459	DP-L4077 + *yjbH*::*Tn*	This study
DP-L6460	DP-L4077 + *P-spxA1*::*Tn*	This study
DP-L6461	DP-L4077 + *ohrA*::*Tn*	This study
DP-L6462	DP-L4077 + *arpJ*::*Tn*	This study
DP-L6463	DP-L4077 + *pplA*::*Tn*	This study
DP-L6464	DP-L4077 + *gshF*::*Tn*	This study
DP-L6189	Δ*gshF* pPL2.*gshF*.*His*	[17]
DP-L6480	*yjbH*::*Tn* pPL2.*yjbH*.*His*	This study
DP-L6481	*P-spxA1*::*Tn* pPL2.*spx*.*His*	This study
DP-L6482	*arpJ*::*Tn* pPL2.*arpJ* region	This study
DP-L6483	*ohrA*::*Tn* pPL2.*ohrRA*	This study
DP-L6507	*ΔyjbH*	This study
DP-L6508	pPL2.*actA1p-TagRFP*	This study
DP-L6509	Δ*yjbH* pPL2.*actA1p-TagRFP*	This study

Knock-in of pPL2 derivative plasmids was performed by standard methods [41]. Briefly, constructed pPL2 plasmids were transformed into chemically competent SM10 *E*. *coli*, selecting on chloramphenicol. Donor SM10 and recipient *L*. *monocytogenes* were mixed at a 1:1 ratio on a non-selective BHI plate at 37°C for 4–24 hours, then trans-conjugation was selected for by plating bacteria on BHI containing streptomycin plus either chloramphenicol (pPL2), erythromycin (pPL2e), or tetracycline (pPL2t). Single colonies were re-streaked for purifying selection onto BHI containing the same antibiotics as used after trans-conjugation.

In-frame deletions of genes was accomplished by allelic exchange using pKSV7-oriT and conventional methods [64]. Briefly, the constructed knock-out plasmid was transformed into SM10 *E*. *coli*, recovered on LB containing carbenicillin, and trans-conjugated into *L*. *monocytogenes* by mixing the donor SM10 and recipient *L*. *monocytogenes* at a 1:1 ratio on a non-selective BHI plate for 4–24 hours at 30°C, the permissive temperature for pKSV7-oriT to replicate in Gram-positive organisms. Trans-conjugation was selected on BHI containing both streptomycin and chloramphenicol at 30°C. After isolation of a single colony of *L*. *monocytogenes* containing pKSV7-oriT at 30°C, bacteria were grown at 42°C on BHI agar containing both streptomycin and chloramphenicol to select for chromosomal integration. Colonies were re-streaked onto selective media at 42°C two additional times for purifying selection and integrated pKSV7-oriT. This strain was then serially passaged at 30°C to enrich for excision and loss of pKSV7-oriT. Mutants that lost pKSV7-oriT were identified by sensitivity to chloramphenicol using indirect patch-plating methods. Finally, allelic exchange was confirmed by PCR and, when necessary, Sanger DNA sequencing.

### *Himar1* mutagenesis and transposon junction sequencing

Preparation of electro-competent *L*. *monocytogenes* and *himar1* transposon mutagenesis were performed as previously described [29], generating a transposon mutant library that was not fully characterized previously [17]. Transposon junctions were mapped as previously described [71]. The position of each *himar1* transposon refers to to the distance of the insertion site, 3’ of the first nucleotide of each gene. Transposons were mapped to the 10403S genome, however, for continuity of nomenclature the EGD-e loci names have been used. For reference: *lmo0441 (LMRG_00133)*, *lmo0443 (LMRG_00135)*, *rsbX* is *lmo0896 (LMRG_02320)*, *yjbH* is *lmo0964 (LMRG_02063)*, *citC* is *lmo1566 (LMRG_01401)*, *lmo2107* is *(LMRG_01261)*, *spxA1* is *lmo2191 (LMRG_01641)*, *ohrA* is *lmo2199 (LMRG_01633)*, *arpJ* is *lmo2250 (LMRG_01581)*, *gtcA* is *lmo2549 (LMRG_01698)*, *pplA* is *lmo2637 (LMRG_02182)*, *gshF* is *lmo2770 (LMRG_01925)*.

### Generalized transduction

Transposons in the chromosome were introduced into different genetic backgrounds by generalized transduction using the phage U153, as previously described [29,75]. Briefly, a transducing lysate was generated by lysogenizing approximately 10^9^ CFU of *L*. *monocytogenes* transposon donor with approximately 10^7^ PFU of phage in 3–4 mL of 0.7% LB Agar containing MgSO_4_ and CaCl_2_ (10 mM each) on LB agar and incubated overnight at 30°C. Phage was soaked out of the agar by incubating with 5 mL of TM buffer (10 mM Tris, pH 7.5 and 10 mM MgSO_4_) for 8–24 hours and these recovered phage stocks were filter sterilized. With the newly generated transducing lysate, 10^8^ *L*. *monocytogenes* recipients were lysogenized with 10^7^ PFU of lysate, incubated at 30°C for 30 min in LB containing MgSO_4_ and CaCl_2_ (10 mM each), and then plated on selective BHI agar at 37°C. When transducing the *himar1* transposon using erythromycin selection, colonies appeared after two days. These colonies were purified by re-streaking transductants for single colonies and verified by sequencing the transposon junction. U153 phage stocks were propagated using *L*. *monocytogenes* strain SLCC-5762.

### Cloning and plasmid construction

Knock-in plasmids were constructed as previously described using primers listed in Table 4 and reagents are from New England Biolabs, unless otherwise specified [71]. Briefly, vectors for complementing *yjbH* and *spxA1* were constructed by amplifying each gene along with its predicted native promoters using a reverse primer that appended a DNA sequence encoding a six histidine affinity tag at the C-terminus. These DNA fragments and pPL2 [41] were then digested with KpnI and BamHI and ligated using Quick Ligase, according to manufacturer’s instructions. The *arpJ* and *ohrA* complement vectors were constructed by amplifying their entire predicted operon and predicted native promoter (*arpJ*: *LMRG_01581-LMRG_01580*, *ohrA*: *LMRG_01632-LMRG_01633*) without addition of affinity tags. The DNA fragment was combined with linearized pPL2t harboring a transcriptional terminator [71] and assembled using In-Fusion Cloning (Clontech) or Gibson Assembly Ultra (Synthetic Genomics). The pPL2t.*P*_*hyper*_ *-actA* vector was constructed by amplifying both 5’ UTR and CDS of *actA* (*LMRG_02626*), and combining the DNA fragment with linearized pPL2t harboring a modified *Pspac-hy* (*P*_*hyper*_) [38] sequence: “aattgtgagcgctcacaattttgcaaaaagttgttgactttatctacaaggtgtggcataatgtgtGTAATTGTGAGCGCTCACAATT”, inserted via gBLOCK (IDT), and a transcriptional terminator for assembly using In-Fusion Cloning (Clontech).

**Table 4:** Oligonucleotide primers used in this study.

Target Gene	Forward Primer Sequence^a^	Reverse Primer Sequence^a^
*16S rRNA*	acccttgattttagttgccag	tgtgtagcccaggtcataag
*actA*	cgacataatatttgcagcgac	tgctttcaacattgctattagg
*lmo0443*	ggtgtagttgcagttataggt	tcaagctgtctgatcggcc
*yjbH*	cgatccagcttgtgatgact	gcggctttgactgcaagac
*citC*	ggcattcgttcactaaacgtt	cgattctatgctaccttcttta
*spxA1*	gccgaaaagctcgtgcatg	ccatcctcagtcatacgaag
*ohrA*	ggtgaagttcattcgccaga	cagttgctgttactgtgctc
*arpJ*	ggttcagaagtagtttccct	gtggaacctttcggcattgc
*pplA*	cgacgacaaaggctggaaag	gattgatttttaactaaagaatcg
*gshF*	gaccctaatctccggaagc	tacagagtcaatcgagtccg
pPL2.*yjbH*.*His*	ggccggtaccgatacttttatagcaaaaagaca	ggccggatccttaatgatgatgatgatgatgtaagtttccgatgtatttccag
pPL2.*spxA1*.*His*	ggccggtaccgaaaacatcaatcagagttaaatt	ggccggatccttaatgatgatgatgatgatggttaaccattttttgcgcttca
pPL2t.*arpJ (LMRG_01581-LMRG_01580)*	gctggtaccgggccctaactgttagagccttgcttatg	ccagcttgcggccgcgtataattagctccttttttctataagtgc
pPL2t.*P*_*hyper*_ *-actA*	gtaattgtgagcgctcacaattctgcagaattcatgaatattttttcttatattagctaattaagaag	gaattgtggatggctccagcttgcggccgcttaattattttttcttaattgaataattttgataaacgc
pPL2t.*ohrRA (LMRG_01632-LMRG_01633)*	agggaacaaaagctggtaccggctaaaatataatcaaaagccttac	gtggatggctccagcttgcggccgccttggccgtaaacgcag
pKSV7.*ΔyjbH* 5’ homology	GAGGAGggtaccgtttagaaaaagaagctttggagg	taaattttggttaatcatttgctatcacctgattttcaaattc
pKSV7.*ΔyjbH* 3’ homology	atgattaaccaaaatttaTACATCGGAAACTTATAAaaaagaagcacccattcctg	gaggagctgcagcaccaaaagtagagttttaagcc

^a^ Oligonucleotide primers listed 5’-3’, underline indicates restriction endonuclease site or complementary overhang for Gibson Assembly.

The *pKSV7-oriT*-Δ*yjbH* vector was constructed according to methods previously described [71]. Briefly, the vector was constructed by sequentially amplifying ~1 kb of homology flanking the *yjbH* coding region using primers in Table 3. These two fragments were joined by sequence overlap extension PCR, which included the coding region for the first six and last six amino acids of YjbH. The final PCR fragment and *pKSV7-oriT* were digested with KpnI and PstI (rSAP was also included for the vector) and ligated using Quick Ligase. The ligation product was transformed into XL1 Blue *E*. *coli* and transformants were screened by PCR for the presence of the insert, followed by Sanger sequencing confirmation.

### Plaque assay

The plaque assay was carried out by conventional methods [22,76]. Briefly, L2 fibroblasts (generated previously from L929 cells [77] and provided as a generous gift from Susan Weiss in 1988, as detailed in Sun et al. [22]) or TIB-73 hepatocytes (ATCC TIB-73) were maintained in high-glucose DMEM medium plus 10% FBS (Hyclone), 2 mM L-glutamine (Gibco), and 1 mM sodium pyruvate (Gibco). Cells were plated at 1.2 x 10^6^ cells per well in a six-well dish and infected the next day at an MOI of 300 with *L*. *monocytogenes* grown overnight at 30°C, stationary. The infection was allowed to proceed for one hour before the wells were washed twice with PBS and 3 mL of medium plus 0.7% agarose and 10 μg/mL gentamicin was overlaid. At 48 hours post-infection the plaques were stained with 2 mL of medium plus 0.7% agarose, 10 μg/mL gentamicin, and 25 μL/mL neutral red (Sigma). The plaques were then imaged at 72 hours post-infection. Plaque area was quantified using ImageJ software [78]. Each experiment represented an average of the area of at least five plaques per strain as a proportion to wild type plaques in that experiment. Data are representative of at least three experiments.

### Macrophage growth curves

Macrophage growth curves were performed as previously described [72,79]. Briefly, bone marrow-derived macrophages (BMMs) were derived from bone marrow of C57BL/6 mice purchased from The Jackson Laboratory and were cultivated/differentiated in high-glucose DMEM medium containing CSF (from mouse CSF-1-producing 3T3 cells), 20% FBS (Hyclone), 2 mM L-glutamine (Gibco), 1 mM sodium pyruvate (Gibco), and 14 mM 2-mercaptoethanol (BME, Gibco). BMMs were derived as previously described and plated in 60 mm non-TC treated dishes that contained 14 TC-treated coverslips at 3 x 10^6^ cells per dish. These dishes were then infected at an MOI of 0.1 for 30 minutes, washed twice with PBS prior to replacing media, and gentamicin was added at 50 μg/mL one hour post-infection. Three coverslips were removed from each dish at 0.5, 2, 5, and 8 hours post-infection and added to 5 mL of sterile water. Coverslips were rigorously mixed prior to plating on LB agar. Each graph is representative of three experiments and each data point represents the average of three coverslips.

### Virulence assays and *in vivo* suppressor analysis

To analyze virulence, female CD-1 mice were infected intravenously (i.v.) via the tail vein using 200 μL of sterile PBS containing 10^5^ CFU of each *L*. *monocytogenes* strain as previously described [80]. The infection was allowed to progress for 48 hours, at which point animals were euthanized and the spleens and livers were harvested. Organs were homogenized in 0.1% NP-40 and serial dilutions were plated on LB agar containing streptomycin. Graphs represent pooled data from at least two experiments of greater than four mice each. Groups were statistically compared using a heteroscedastic Student’s *t-test*.

*In vivo* suppressors were identified similarly to previously described methods [17]. Briefly, CD-1 mice were infected i.v. with 1 x 10^7^ CFU of Δ*gshF* for 72 hours and the livers were harvested, homogenized, and 100 μL was inoculated into broth. Naïve mice were then infected with these liver homogenate cultures. After four successive infections bacteria isolated from infected livers were analyzed via plaque assay and two strains with intermediate plaque phenotype were selected for genome sequencing.

### Genome sequencing

Genomic DNA was isolated from *L*. *monocytogenes* using the MasterPure Gram-Positive DNA Purification Kit (Epicentre) according to the manufacturer's instructions. Genome sequencing and DNA library preparation was performed as previously described [71] at the Vincent J. Coates Genomics Sequencing Laboratory at UC Berkeley. Data was assembled and aligned to the 10403S reference genome (GenBank: GCA_000168695.2) demonstrating >50x coverage. SNP/InDel/structural variation was determined as compared to the Δ*gshF* parent strain using CLC Genomics Workbench (CLC bio).

### Immunoblots

All immunoblotting was performed as previously described [17]. Briefly, for bacteria grown in broth, overnight cultures were diluted 1:10 into BHI, incubated for five hours at 37°C, shaking, then the bacteria were separated from the supernatant by centrifugation. For secreted proteins, the supernatant was treated with 10% v/v TCA for one hour on ice to precipitate all proteins. The protein pellet was washed twice with ice- cold acetone, followed by vacuum drying. The proteins were dissolved in LDS buffer (Invitrogen) containing 5% BME using a volume that normalized for OD_600_ of harvested bacteria, boiled for 20 minutes, and separated by SDS-PAGE. For surface associated proteins, bacteria were suspended in 150 μL of LDS buffer containing 5% BME, boiled for 20 minutes, and proteins separated by SDS-PAGE.

Immunoblots of bacteria grown intracellularly within infected BMMs used 12-well dishes with BMMs at a density of 10^6^ cells per well and infected with an MOI of 10. One hour post-infection the cells were washed and media containing gentamicin (50 μg/mL) was added. Four hours post-infection the cells were washed twice with PBS and harvested in 150 μL LDS buffer containing 5% BME. The samples were then boiled and separated by SDS-PAGE. Primary antibodies were each used at a dilution of 1:5,000, including: rabbit polyclonal antibody against the N-terminus of ActA [81], rabbit polyclonal antibody against LLO, and a mouse monoclonal antibody against P60 (Adipogen). P60 is a constitutively expressed bacterial protein used as a loading control [82]. All immunoblots were visualized and quantified using Odyssey Imager and appropriate secondary antibodies from the manufacturer according the manufacturer’s instructions.

### Quantitative RT-PCR of bacterial transcripts

Transcript analysis in broth was performed as previously described [83]. Briefly, bacteria were grown overnight in BHI and subcultured 1:100 into 25 mL BHI. Bacteria were harvested at an OD_600_ = 1.0. Transcript analysis during infection was analyzed as previously described [17]. Briefly, BMMs were plated at a density of 3 x 10^7^ cells in 150 mm TC-treated dishes and infected with an MOI of 10. One hour post-infection the cells were washed and media containing gentamicin (50 μg/mL) was added. Four hours post-infection the cells were washed with PBS and lysed in 5 mL of 0.1% NP-40. After collecting the lysate, the dishes were then washed in RNAprotect Bacteria Reagent (Qiagen), which was combined with the lysate. Bacteria were isolated by centrifugation. Bacteria harvested from either broth or BMMs were lysed in phenol:chloroform containing 1% SDS by vortexing with 0.1 mm diameter silica/zirconium beads (BioSpec Products Inc.). Nucleic acids were precipitated from the aqueous fraction overnight at -80°C in ethanol containing 150 mM sodium acetate (pH 5.2). Precipitated nucleic acids were washed with ethanol and treated with TURBO DNase per manufacturer’s specifications (Life Technologies Corporation). RNA was again precipitated overnight and then washed in ethanol. RT-PCR was performed with iScript Reverse Transcriptase (Bio-Rad) and quantitative PCR (qPCR) of resulting cDNA was performed with KAPA SYBR Fast (Kapa Biosystems). Primers used for qPCR are listed in Table 4.

### Disk diffusions

Disk diffusions were performed similarly to previously described methods [84]. Briefly, approximately 3 x 10^7^ CFU from overnight cultures of bacteria were immobilized using 4 mL of molten (55°C) top-agar (0.8% NaCl and 0.8% bacto-agar) spread evenly on tryptic soy agar plates. After the agar cooled, Whatman paper disks soaked in 5 μL of 5% hydrogen peroxide, 1 M diamide solution, or 80% cumene hydroperoxide solution were placed on top of the bacteria-agar. The zone of inhibition was measured after 18–20 hours of incubation at 37°C.

### *actA1p-rfp* fluorescence measurements in BMMs

BMMs were differentiated and cultivated as described for BMM growth curves. Cells were plated at 5 x 10^5^ cells per well in a 24-well dish in media without antibiotics. The following day BMMs were infected at an MOI of 5 with *L*. *monocytogenes* mutants that had been incubated at 30°C without shaking. After 30 minutes cells were washed once with PBS and fresh media containing gentamicin (50 μg/mL) was added. Six hours post infection media was removed from each well, the cells were washed with 1 mL of PBS, and 0.5 mL of PBS was replaced for each well. RFP fluorescence was measured using a plate reader (Infinite M1000 PRO, TECAN) with 555 nm excitation, 584 nm emission, and 5 nm band filters. Each well was interrogated 64 times on an 8 X 8 grid and the edge reads were excluded. Data were normalized by subtracting baseline fluorescence of wild type (without RFP) infected cells and plotting data as a percentage of wild type expressing *actA1p-rfp*. Each experiment represents three infected wells per *L*. *monocytogenes* genotype and data are representative of three pooled independent experiments.

## Supporting Information

S1 Fig Analysis of *P-spxA1*::*Tn*. Quantitative RT-PCR of *spxA1* transcript in wild type compared to *P-spxA1*::*Tn* grown in broth. Data are the mean ± s.e.m. of at least three independent experiments and the *p* value was calculated using a heteroscedastic Student’s *t*-test; *** *p* < 0.001.
(TIF)

S2 Fig Complementation of transposon mutants. Plaque area as a percentage of wild type. Data are the mean ± s.e.m. of at least three independent experiments. Details of each complement strain can be found in the materials and methods.
(TIF)

S3 Fig Growth curve in NOS2^-/-^ and NOX2^-/-^ BMMs. Data indicate the mean ± s.e.m. of data pooled from two independent experiments, each containing three technical replicates.
(TIF)
