# Supplementary figures and images for: An In Vivo Selection Identifies Listeria monocytogenes Genes Required to Sense the Intracellular Environment and Activate Virulence Factor Expression
Source: PLoS Pathog. 2016 Jul 14;12(7):e1005741. doi: 10.1371/journal.ppat.1005741 (PMC4945081; doi:10.1371/journal.ppat.1005741)

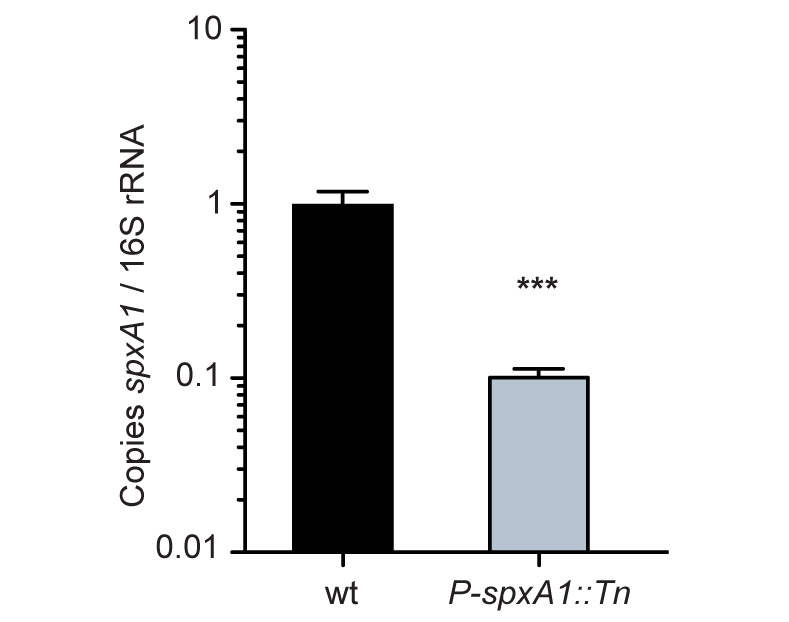

Supplement: S1 Fig — Quantitative RT-PCR of spxA1 transcript in wild type compared to P-spxA1::Tn grown in broth. Data are the mean ± s.e.m. of at least three independent experiments and the p value was calculated using a heteroscedastic Student’s t-test; *** p < 0.001. (TIF) [file ppat.1005741.s001.tif]

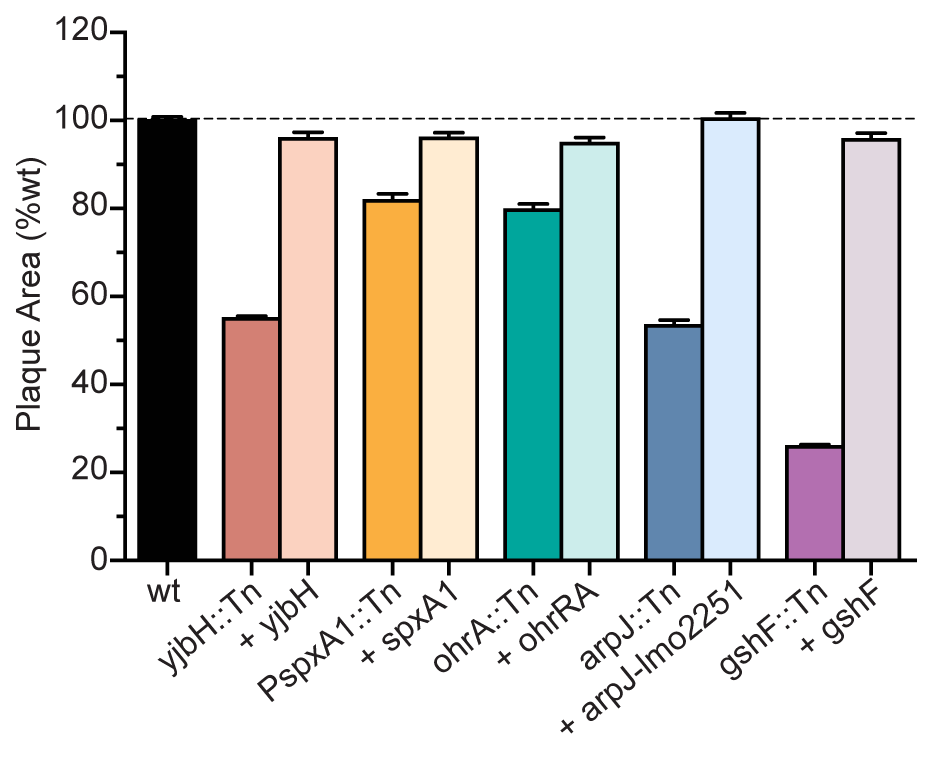

Supplement: S2 Fig — Plaque area as a percentage of wild type. Data are the mean ± s.e.m. of at least three independent experiments. Details of each complement strain can be found in the materials and methods. (TIF) [file ppat.1005741.s002.tif]

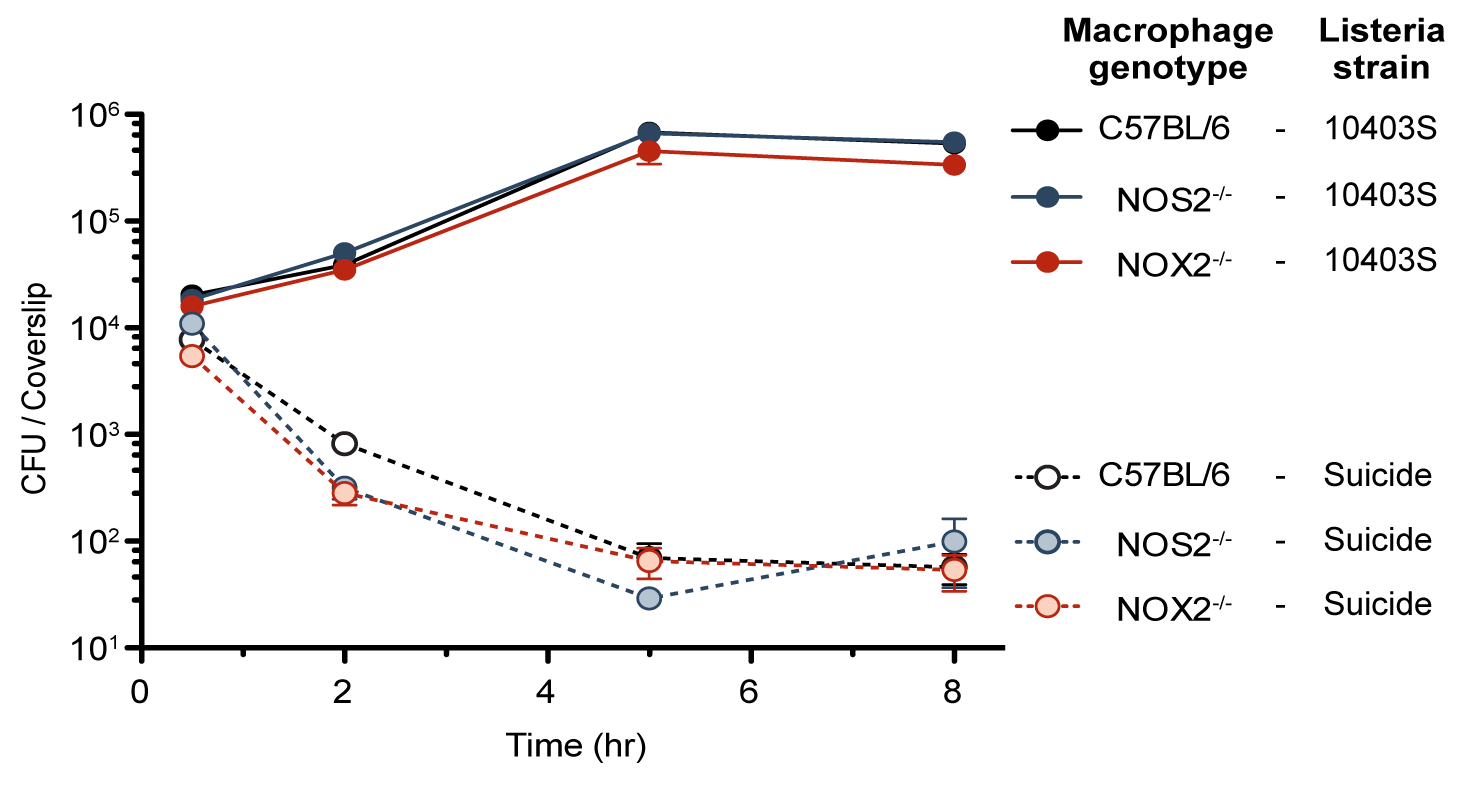

Supplement: S3 Fig — Data indicate the mean ± s.e.m. of data pooled from two independent experiments, each containing three technical replicates. (TIF) [file ppat.1005741.s003.tif]
